# Nanoscale visualization of crack tips inside molten corium–concrete interaction debris using 3D-FIB-SEM with multiphase positional misalignment correction

**DOI:** 10.1093/jmicro/dfaf005

**Published:** 2025-01-25

**Authors:** Hotaka Miyata, Kenta Yoshida, Kenji Konashi, Yufeng Du, Toru Kitagaki, Takahisa Shobu, Yusuke Shimada

**Affiliations:** Institute for Materials Research, Tohoku University, 2145-2 Naritacho, Oarai, Ibaraki 311-1313, Japan; Institute for Materials Research, Tohoku University, 2145-2 Naritacho, Oarai, Ibaraki 311-1313, Japan; Institute for Materials Research, Tohoku University, 2145-2 Naritacho, Oarai, Ibaraki 311-1313, Japan; National Key Laboratory of Nuclear Reactor Technology, Nuclear Power Institute of China, Changshun Road No.328, Shuangliu District, Chengdu, Sichuan 610213, China; Collaborative Laboratories for Advanced Decommissioning Science, Japan Atomic Energy Agency, 2-4 Shirakata, Tokai-mura, Ibaraki 319-1195, Japan; Materials Sciences Research Center, Japan Atomic Energy Agency, 2-4 Shirakata, Tokai-mura, Ibaraki 319-1195, Japan; Department of Advanced Materials Science and Engineering, Faculty of Engineering, Kyushu University, 744 Motooka, Nishi-ku, Fukuoka 819-0395, Japan

**Keywords:** FIB, SEM, fuel debris, crack, 3D analysis

## Abstract

Characterizing molten corium–concrete interaction (MCCI) fuel debris in Fukushima reactors is essential to develop efficient methods for its removal. To enhance the accuracy of microscopic observation and focused ion beam microsampling of MCCI fuel debris, we developed a 3D focused ion beam scanning electron microscopy technique with a multiphase positional misalignment correction method. This system automatically aligns voxel positions, corrects contrast and removes artifacts from a series of over 500 scanning electron microscopy images. The multiphase positional misalignment correction method, which focuses on time-modulated contrast, considerably reduces charge-up artifacts in glass phases, enabling 3D morphological observation and analytical transmission electron microscopy of crack tips in two types of MCCI debris at the 3D/nanoscale for the first time. In the Fe–ZrSiO_4_-based debris, metallic balls composed of Fe, Cr_2_O_3_ and ZrO_2_ with dimples on the surface of about 2–58 µm in diameter were observed at the crack tips. In the (Zr, U)SiO_4_-based debris, a core–shell structure composed of a (U, Zr)O_2_ core with a diameter of about 1–5 μm and a (Zr, U)SiO_4_ shell with a diameter of about 2–9 μm in complex MCCI fuel debris at the crack tips.

## Graphical Abstract

**Figure:**
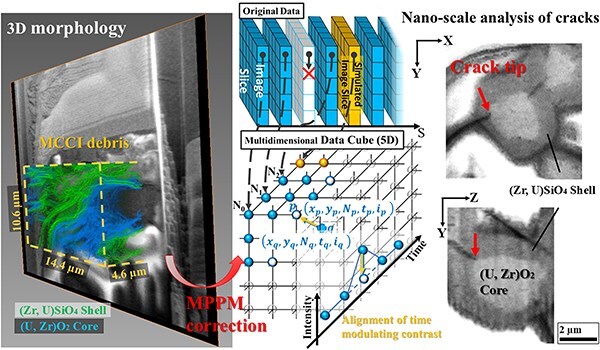

## Introduction

**Fig. 1.**
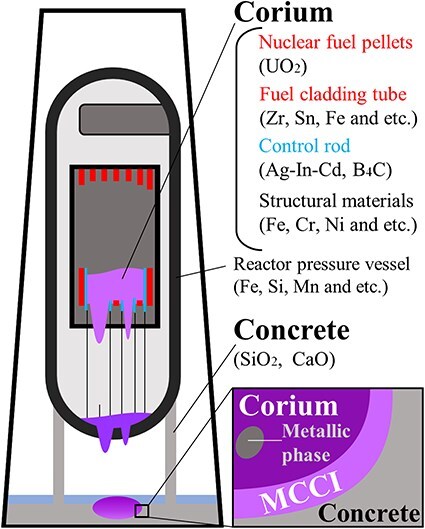
Schematic of MCCI debris assumed to exist in the Fukushima nuclear power reactor. MCCI, molten corium–concrete interaction.

Along with the Chernobyl and Three Mile Island accidents, the serious accident occurring at the Fukushima Daiichi power plant in Japan provided useful knowledge from which safer nuclear energy policies have been established. Figure 1 shows a schematic of the molten corium–concrete interaction (MCCI) fuel debris that is assumed to be present in the Fukushima reactors [1]. Previous studies have shown that molten nuclear fuel (UO_2_) reacts with fuel cladding (Zr) structural material (FeCrNi) and concrete silica to form (U, Zr)O_2_ and the zircon material (Zr, U)SiO_4_ [2], which are multiphase MCCI debris that form microscopic vitrified bodies of ductile metals and hard oxides embedded in soft glass. Some reports suggest that this degradation of the glass quality, which causes crack formation and embrittlement due to neutron irradiation, contributes to increasing the air dose rates around the Chernobyl sarcophagus [3].

To improve the accuracy of microscopic measurements for MCCI fuel debris, we investigated recent advancements in serial sectioning 3D focused ion beam scanning electron microscopy (3D-FIB-SEM) [4,5]. 3D-FIB-SEM, which allows polishing and performing surface analysis of radioactive isotope samples in a high vacuum chamber, is a suitable method for observing the morphology of MCCI debris. This 3D-FIB-SEM can be effectively applied to Fukushima MCCI debris containing nuclear species, such as europium and radon derived from uranium fuel, and enables microsampling for analytical transmission electron microscopy (TEM) in volumes as small as 500 μm^3^. 3D-FIB-SEM was applied to fuel debris by Miller *et al*. in 2018 [6]. Similar FIB sampling and TEM analyses of extremely rare micro-volume samples, such as meteorites were conducted by Kimura *et al*. [7].

To further improve the accuracy of 3D-FIB-SEM analysis of complex MCCI debris, we redesigned the reconstruction method. Given the lack of software developed for unique cases like Fukushima MCCI debris, we developed a multiphase positional misalignment (MPPM) correction method capable of simultaneously addressing the sample drift [8,9] and the charge-up artifacts [10,11] caused by glassy regions. The MPPM correction is based on the two-stage alignment method proposed by Iryna Reimer for 3D-FIB-SEM observation of known mineral structures [12]. In our Python-based MPPM correction program, the original 3D data are expanded into a 5D data space for processing, enabling the removal of microscopic imaging noises, image distortion and scanning noise, as well as time-modulated artifacts.

The 3D-FIB-SEM technique is expected to enhance both the reproducibility and accuracy of TEM analysis of complex debris. This technique was performed in conjunction with conventional microstructural evaluations using weak-beam scanning transmission electron microscopy (WB-STEM) [13] and scanning transmission electron microscopy–energy dispersive X-ray spectroscopy (STEM–EDS) [14] analyses as part of the decommissioning process of the Fukushima debris.

## Experimental procedure

### MCCI debris sample preparation

As MCCI debris samples, Fe–ZrSiO_4_-based debris and (Zr, U)SiO_4_-fuel debris were synthesized using the following equipment and procedures. ZrO_2_ and UO_2_ powders were mixed using a ball mill and then compressed into pellets using a compression machine. The as-obtained pellets were sintered at 1988 K in an electric furnace for 6 h to form a solid solution of (U, Zr)O_2_, which was ground using a tungsten mortar. Then, the (U, Zr)O_2_ powder or ZrO_2_ powder was mixed with steel use stainless (SUS), SiO_2_ (64.7 wt%), CaO (13.2 wt%), Al_2_O_3_ (16.0 wt%) and Fe_2_O_3_ (6.1 wt%) powders with compositions simulating those of the fuel debris in Nuclear reactor unit 1 at the Fukushima Daiichi Nuclear Power Station (Fig. 1). The mixed powder was placed in a platinum (Pt) pan of 5 mm diameter and heated at 1673 K for 5 h under an argon atmosphere. The Fe–ZrSiO_4_-based debris model is used to study metal–oxide interfaces resulting from zircaloy fuel cladding and reactor structural materials [1–3,15]. The (Zr, U)SiO_4_-fuel debris model is used to investigate the ZrSiO_4_–UO_2_ interface in MCCI debris.

**Fig. 2.**
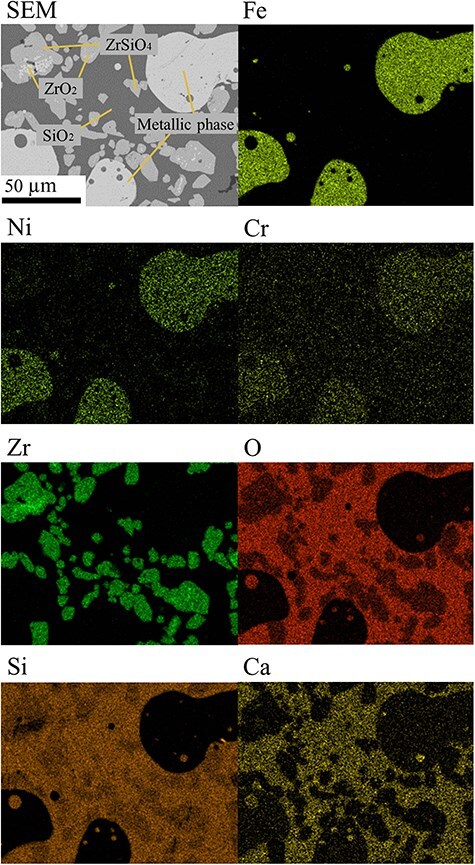
Microtome section of the Fe–ZrSiO_4_-based debris: SEM image and EDS elemental mapping. SEM, scanning electron microscopy; EDS, energy dispersive X-ray spectroscopy.

Figure 2 shows SEM images and SEM–EDS mappings of microtome sections of the Fe–ZrSiO_4_-based MCCI debris, which demonstrate that homogeneous MCCI debris with few voids was created using a small Pt pan of 5 mm diameter. For the 3D-FIB-SEM observation of the crack tips, cracks at the interface between the Pt pan and MCCI debris stemming from the difference in thermal expansion coefficient were used as an indicator.

### 3D reconstruction and TEM analyses of crack tips in MCCI debris

**Fig. 3.**
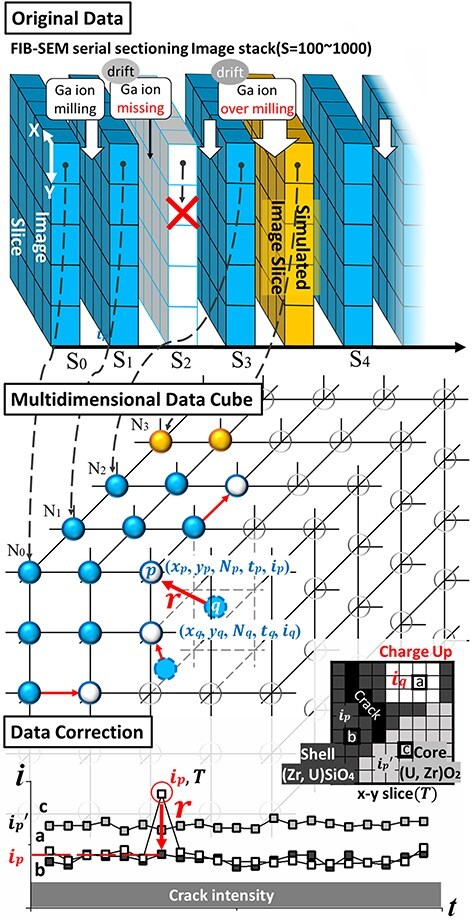
Conceptual diagram of the MPPM correction method. MPPM, multiphase positional misalignment.

Figure 3 shows a schematic of the MPPM correction method developed in the present study for an accurate reconstruction of the debris complex structure. The original idea was previously reported by Iryna Reimers for the 3D reconstruction of various rock samples [12]. In the MPPM correction method, a function is used for drift correction along a slice direction (*Z*-direction) and another function is adopted to determine and remove microscopic artifact pixel intensities such as those caused by charge-up. This method expands the 3D data from the original SEM image into a 5D data space for processing, enabling the removal of noise related to microscope imaging such as image distortion and scanning noise along with time-modulated artifacts.

Correction of misalignment in the *Z*-direction (stack direction) is extremely important in 3D reconstruction; in TEM serial section observation, an alignment method based on the known structure is widely used [16,17]. Recently, advanced alignment methods combining computed image processing and statistical discriminant analysis circuits have been proposed [18,19]. In the case of the 3D-FIB-SEM reconstruction, the accurate correction of misalignment in the *Z*-direction was achieved by developing a specimen processing method to form specific fiducial markers [20,21] and a special equipment to detect the *Z*-directional misalignment [22]. However, such misalignment corrections generally focus on the image shift along the *x*- and *y*-directions [23].

In the MPPM correction method, the original data are first stored in the data cube after drift determination in the *Z*-direction. If the positive *Z*-drift is larger than the slice pitch (40 nm) and gallium (Ga) ion irradiation is lost, storage is postponed; conversely, if the *Z*-drift is negative, it is supplemented with simulated data. General drift correction and noise processing are performed in this multidimensional data cube, which contains five variables in this case, i.e. *x*, *y*, *N*, *t* and *i*. The MPPM algorithm, which comprises both the drift correction phase and contrast correction phase, calculates the displacement vector ***r*** in the 5D data space and moves point ***p*** to point ***q***. The drift correction phase achieves an accuracy of 2.5–3.0 nm, which is approximately one-tenth of *Z*-direction slice pitch. The contrast correction phase determines the judgement range of 500 s to remove the intensity *i*_p_, corresponding to *N* slices of 5p before and after (if the *N*-axis and the original data *S*-axis are parallel, the judgement occurs in the *Z*-direction over 300 nm of depth range. Threshold values for permitted intensity changes (10p/*t*) and crack judgement (35p) allow for voxel reconstruction in regions such as the (i) charge-up region, (ii) (Zr, U)SiO_4_ shell region and (iii) (U, Zr)O_2_ region, as schematically depicted in Fig. 3. These reconstructions are based on continuity in contrast. This feature of the MPPM correction allows identifying time-modulating artifacts such as those due to charge-up and scan noise and restoring only the intensity of the target data cube element. It also supports iterative correction. The first microscopic breakthrough in this study is the high-contrast 3D rendering of cracks in vitrified glass, which were previously completely invisible in *Z*-directional average processing, by means of the unique MPPM correction.

The MPPM correction is an innovative imaging process for nuclear materials and fuel debris that enables the nanoscale 3D analysis of valuable and radioactive samples without requiring equipment with complicated modifications or special geometry and can be adopted to resolve the charge-up artifacts of 3D-FIB-SEM [24, 25]. Our Python-based program for the MPPM correction is available for download at the website, which can be accessed using the following link: http://www.imr-oarai.jp/archive_mppm_microscopy2025/.

3D-FIB-SEM and microsampling of thin-film samples at crack tip were performed using a focused Ga ion beam system (FIB, 30 kV Helios, Thermo Fisher) with milling conditions including an acceleration voltage of 30 kV, a current density of 2.5 nA, a milling target thickness of 50 nm, a processing time of 1.5 min for FIB, an acceleration voltage of 5 kV, a local electron density of 50 pA, a dwell-time of 0.05 µs and an image size 768 × 512 pixels for SEM (425 images, total processing time: 12 h and 40 min). Then, a low-voltage Ar ion beam system (GentleMill 300 V Technorg-Linda) was used to remove Ga ion damage on the top and bottom sides for 1.5 h. Dislocation analysis of stress concentration points at the crack tips and chemical composition analysis were performed using WB-STEM (200 kV ARM JEOL) and an silicon-drift detector (SDD JEOL) with a diameter of 100 mm. The WB-STEM imaging is characterized by freely setting the optimal electron beam convergence and detection angles for specific lattice defects existing inside the material with off-axis diffraction conditions. The STEM imaging of microstructures in nuclear materials has become more popular with the development of 200 kV diffraction contrast imaging scanning transmission electron microscopy (DCI-STEM) [26] and 300 kV diffraction selected on-zone STEM (DsoZ-STEM) [27], which can combine the nanoscale dislocation analysis with atomic resolution of STEM–EDS analyses along zone axes. WB-STEM, DIC-STEM and DsoZ-STEM can be selected depending on the size of the target-irradiated defect aggregate and the spatial resolution of the EDS chemical analysis.

## Results and discussion

**Fig. 4.**
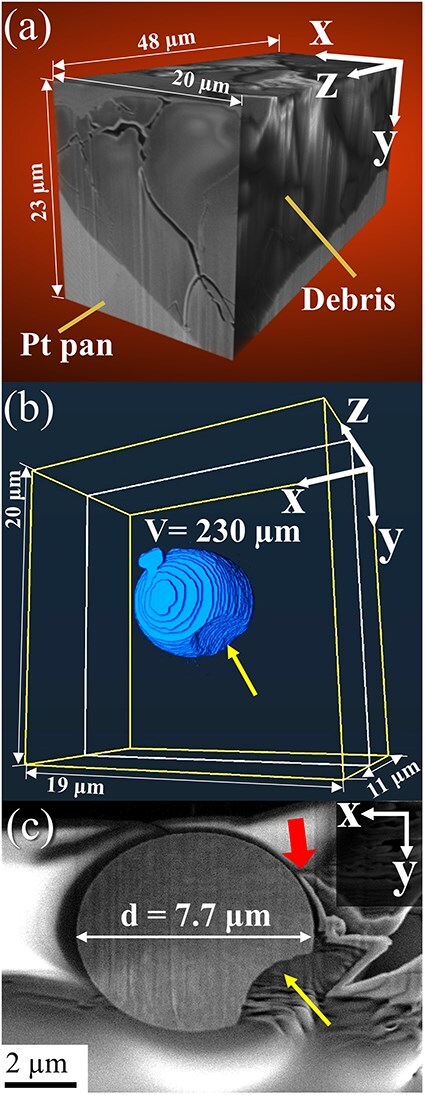
3D-FIB-SEM of the Fe–ZrSiO_4_-based debris. (a) 3D reconstruction of cracks near Pt pan/the Fe–ZrSiO_4_-based debris boundary; (b) 3D reconstruction of the partially melting of the metallic phase (yellow allow) and (c) slice image of the crack (red arrow) and metallic phase–(Zr, U)SiO_4_ interface. 3D-FIB-SEM, 3D focused ion beam scanning electron microscopy.

**Fig. 5.**
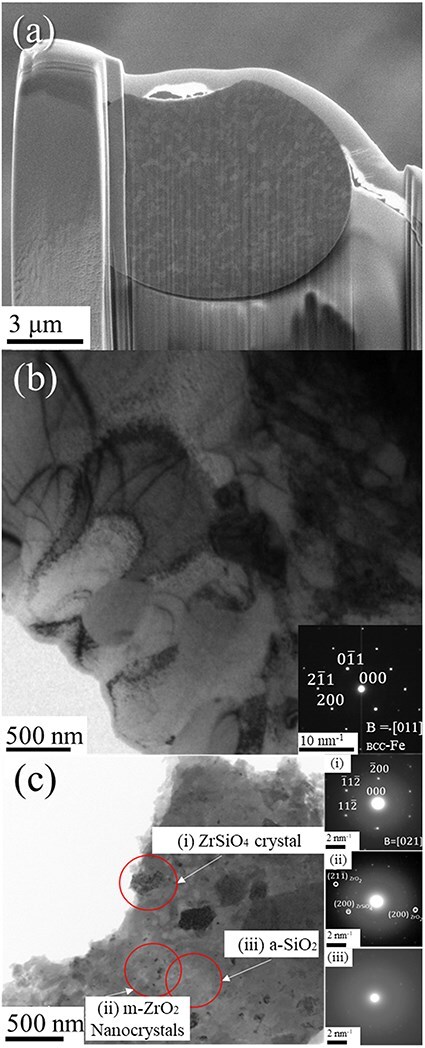
(a) Microsampling of metallic phases using the FIB-SEM system; (b) bright-field image of metallic phases and (c) diffraction patterns of samples prepared using the grinding method: (i) single-crystal ZrSiO_4_, (ii) polycrystalline m-ZrO_2_ and (iii) amorphous SiO_2_. FIB-SEM, focused ion beam scanning electron microscopy.

**Fig. 6.**
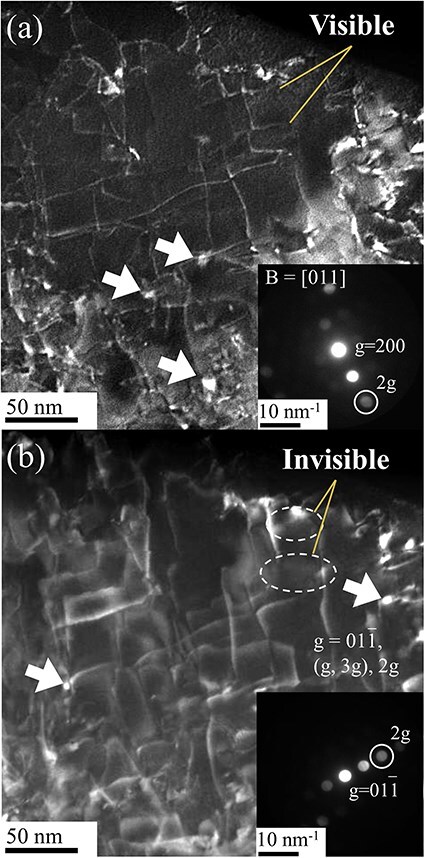
WB-STEM observations of metallic phases with diffraction conditions of (a) g = 200, (g, 3g), 2g and (b) g = 01$\bar 1$, (g, 3g), 2g. WB-STEM, weak-beam scanning transmission electron microscopy.

Figure 4 shows the 3D morphology of the Fe–ZrSiO_4_-based debris reconstructed using the MPPM correction method. Figure 4a reveals cracks formed at the interface between the Pt pan and the debris. Figure 4b shows a surface rendering of metallic phase with an average diameter of 7.7 μm and a total volume of 230 μm^3^. Dimples can be observed on the surface of the metallic phase, indicating that the metallic phase was formed via melting and reprecipitation during the fuel debris sintering process. This means U and radioactive species can be included in the metallic phase [26]. Interestingly, such a metallic phase is present at the tip of more than half of the cracks, as shown in Fig. 4c. Therefore, we analyzed the structure and chemical composition of this metallic phase using a simulated ZrSiO_4_-based fuel debris sample containing no U atoms. Figure 5a and b shows the SEM and bright-field TEM images and diffraction patterns of the metallic phase in the Fe–ZrSiO_4_-based debris sample using the FIB-microsampling technique. The metallic phase was confirmed to be a polycrystalline body composed mainly of body-centered cubic Fe. Figure 5c shows the diffraction patterns of monocrystalline ZrSiO_4_, polycrystalline m-ZrO_2_ and amorphous SiO_2_, which were obtained using the pulverization method in isopropanol solution. These main crystalline phases identified in this study are in agreement with the X-ray diffraction and selected area electron diffraction results of previous studies [28]. In previous studies of fuel debris [29], the metallic phase was treated as a region that did not contain U (Zr in the case of the simulated material), but many dislocations and precipitates were observed in the TEM image, as shown in Fig. 5b. Therefore, in the present study, additional dislocation observation using STEM and chemical composition mapping of the metallic phases were performed as shown in Fig. 6.

Figure 6 shows the determination of the Burgers vector of dislocations using WB-STEM. The diffraction conditions used for dark-field WB-STEM imaging were g = 200, (g, 3g), 2g and g = 01$\overline 1 $, (g, 3g), 2g. The Burgers vector of the dislocation was determined to be ½ [111] or ½ [$\bar 1$11] by the g–b visible/invisible criterion. The dislocation line density was ∼2.1 × 1014 m^−2^. The line density was considerably higher than that of pure Fe 2.0 × 1011 m^−2^ [30] and Fe–0.01%C 7.3 × 1012 m^−2^ [31] after general annealing. The dislocation density suggested a dense dislocation network in martensitic steels, which are generally within the range of 0.6–3.0 × 1015 m^−2^. In addition, a strong contrast on the grains was found in the dark-field WB-STEM image, as indicated by the white arrows. This strong diffraction contrast suggests the presence of a lattice-mismatched precipitate. Sizes of such lattice-mismatched precipitate were estimated to be 5–10 nm in diameter.

**Fig. 7.**
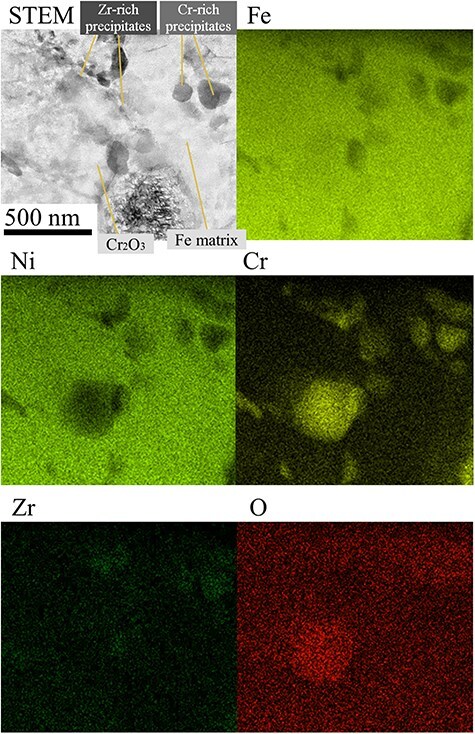
Bright-field STEM image after MPPM contrast correction and STEM–EDS mapping of the metallic phases of the Fe–ZrSiO_4_-based debris. STEM, scanning transmission electron microscopy; MPPM, multiphase positional misalignment; EDS, energy dispersive X-ray spectroscopy.

Figure 7 summarizes the results of the STEM–EDS analysis of the chemical composition of the metallic phases. This elemental mapping was performed using cold field emission dun and EDS detector of 100 mm in a diameter. Bright field STEM image was denoised through the MPPM contrast correction. In this experiment, we focused on O and Zr as simulated U element in addition to Fe, Ni and Cr elements, which are the main components of stainless steel (SUS) powder. As expected, Cr_2_O_3_ was identified as a rich Cr precipitate. In addition, Zr elements were identified inside the metallic phase. Distribution of the Zr element suggested that grain sizes of crystal including Zr elements are very small. STEM–EDS concluded that Zr precipitates synthesized at the interfaces of Fe/Cr_2_O_3_ and Fe grain boundaries, also around dislocations as indicated by white arrows in Fig. 6. These Zr precipitates are reasonably ZrO_2_ nanocrystals, <10 nm in a diameter. The detailed structures of metallic phase are shown in Figs. 5–7 indicate that Fe was in a molten state during the sintering process, in which produces ZrSiO_4_ and when the surface of the raw material ZrO_2_ powder are also partially melted. It should be noted that this is the first time that this phenomenon has been observed in crack tips of the Fe–ZrSiO_4_-based debris. In actual fuel debris, the metallic phase is treated as nuclear fuel contaminant containing UO_2_, which is known to behave very close to ZrO_2_.

However, the confining effect of U elements on the metallic phase of the crack tips is very high. First, the internalized Zr atoms (as simulated U atoms) are small oxides, which are stabilized by forming complexes with very dense dislocations. In our preliminary thermal stability evaluation, both dislocations and precipitates were not deformed at all during *in situ* annealing tests up at about 500°C [32]. The risk of U atoms in a metallic environment leaking into the natural environment is extremely low, unless they are exposed to a corrosive environment of SUS, such as seawater intrusion [33] or acid rain [34]. Therefore, we analyzed the crack tip structure with the highest risk, which necessitates further investigation of the crack tips in the core–shell structure.

**Fig. 8.**
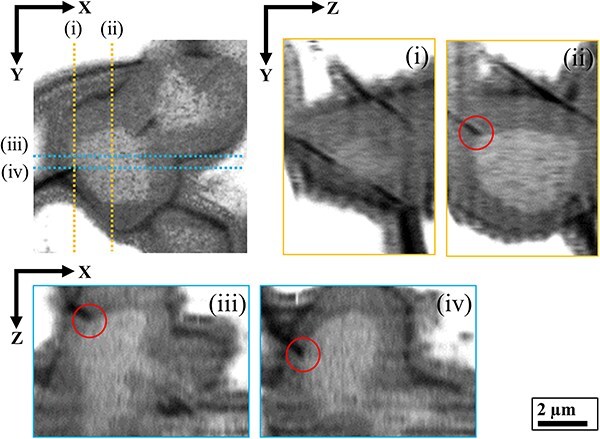
3D-FIB-SEM of the core–shell structure in the (Zr, U)SiO_4_-fuel debris. *XY* cross-sectional slice image with MPPM correction and the corresponding reconstructed *ZY* and *XZ* slice planes (indicated as blue and orange dotted lines in the *XY* slice). 3D-FIB-SEM, 3D focused ion beam scanning electron microscopy; MPPM, multiphase positional misalignment.

We analyzed the crack tip in the core–shell structure with the highest risk in the Fukushima MCCI debris. Figure 8 shows the crack tip structure of the (Zr, U)SiO_4_-fuel debris reconstructed using our MPPM correction method. By performing drift correction and determination and correction of each voxel intensity, microcracks around the UO_2_ core–(Zr, U)SO_4_ shell structure, which were previously completely invisible due to charge-up of the glassy regions, were visualized for the first time. The dotted lines (i)–(iv) in the *XY* slice of the reconstructed tomogram correspond to the *XZ* and *ZY* slice planes, respectively. The red circles in the *XZ* and *ZY* slices (ii), (iii) and (iv) highlight the crack tip, which clearly extends into the UO_2_ core. In this study, we analyzed six core–shell structures in the (Zr, U)SiO_4_-fuel debris using a 3D approach. The structural parameters are summarized in Tables 1 and 2. High-resolution 3D-FIB-SEM with MPPM correction revealed the following findings on the crack tips of MCCI debris. At the crack tips, there were a metallic ball or a bilayer particle consisting of a (U, Zr)O_2_ core and a (Zr, U)SiO_4_ shell. Cracks penetrated the glassy regions and the (Zr, U)SiO_4_ shells but never cut off the (U, Zr)O_2_ core nor the metallic ball; however, they may be connected along the core and ball. Cracks propagated mainly at the boundary between the glassy regions and the (Zr, U)SiO_4_ shells. Well-defined cracks, which are shown in the XY, ZY and XZ slice images in Fig. 8, exhibited a gap of about 500 nm (area with luminosity <35p).

**Table 1.** 3D analysis of the size of the (U, Zr)O_2_ cores in the (Zr, U)SiO_4_-fuel debris

Core No.	Major axis dia. (µm)	Minor axis dia. (µm)	Feret dia. (µm)	Krummbein dia. (µm)
1	4.4	2.9	3.2	3.1
2	5.0	1.6	3.5	3.5
3	3.9	3.1	3.0	2.8
4	3.2	2.1	2.9	2.6
5	1.5	0.8	1.8	1.6
6	3.4	2.1	2.9	2.9

**Table 2.** 3D analysis of the size of the (Zr, U)SiO_4_ shell in the (Zr, U)SiO_4_-fuel debris

Shell No.	Major axis dia. (µm)	Minor axis dia. (µm)	Feret dia. (µm)	Krummbein dia. (µm)
1	6.7	4.4	5.2	5.2
2	8.7	8.0	8.7	8.4
3	4.4	4.1	4.0	3.6
4	7.0	4.9	6.5	6.3
5	4.3	3.8	3.7	3.7
6	3.7	3.1	N. A.	N. A.

**Fig. 9.**
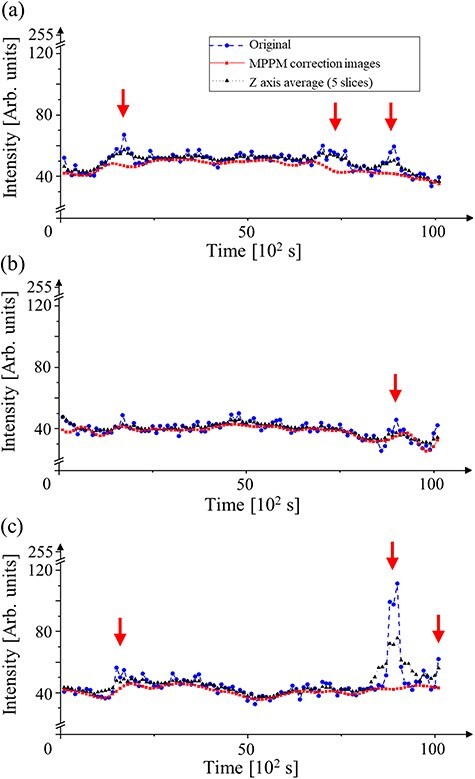
Intensity profiles from tomograms of the core–shell structure in the (Zr, U)SiO_4_-fuel debris at (a) (U, Zr)O_2_ core, (b) and (c) (Zr, U)SiO_4_ shell regions.

**Fig. 10.**
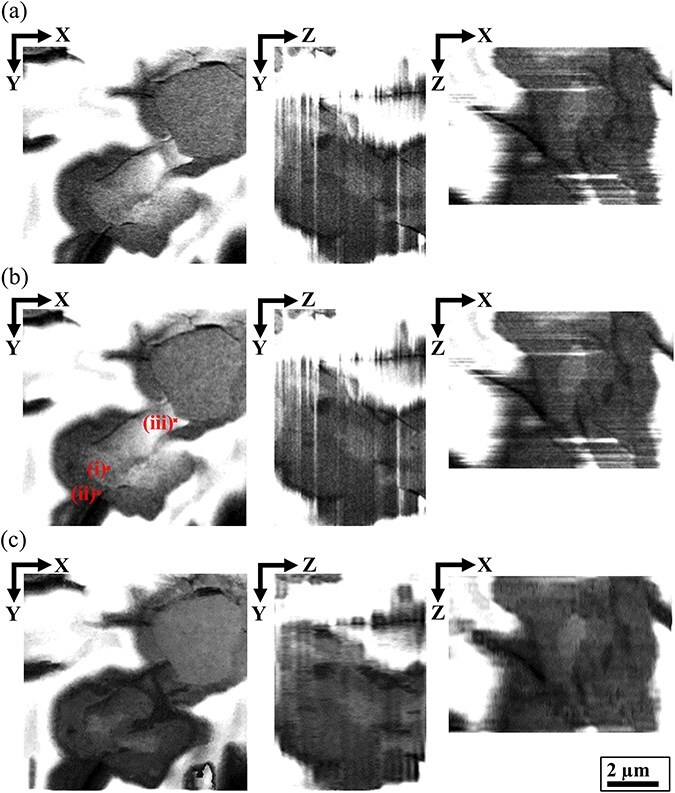
*XY*, *ZY* and *XZ* slices of tomograms of the core–shell structure in the (Zr, U)SiO_4_-fuel debris after (a) conventional image stack, (b) drift MPPM correction and (c) drift-contrast MPPM correction. MPPM, multiphase positional misalignment.

Figure 9 presents the time-modulated intensity profiles corrected for the (U, Zr)O_2_ core region, (Zr, U)SiO_4_ shell region and (Zr, U)SiO_4_ shell region exhibiting considerable charge-up artifacts. The original contrast processing approach along the time axis played a key role in artifact removal from the MCCI debris, offering an improvement over conventional charge-up restoration methods. These conventional methods typically rely on a 2D luminance distribution from a single SEM image [35,36]. The red arrows indicate the peak intensity of charge-up artifacts, which can severely compromise the accuracy of the tomogram when processed using averaging methods such as a Gaussian filtering. A comparison between the original intensity (blue) and MPPM corrected intensity (red) demonstrates that our MPPM correction method successfully removed charge-up artifacts from the SEM images. This was achieved through the use of the 5D data cube in the MPPM correction, which enabled the restoration of areas that would otherwise lack contrast in a single SEM image. The *XY*, *ZY* and *XZ* slices of the tomograms shown in Fig. 10 illustrate the results from the (a) conventional image stack in the 3D-FIB-SEM reconstruction, (b) drift MPPM correction and (c) contrast MPPM correction. The improvements made to the tomogram vs MPPM correction are further detailed in Supplementary data (file name: Movie SD1_slice_series_Fuel_debris_MPPM_correction).

**Fig. 11.**
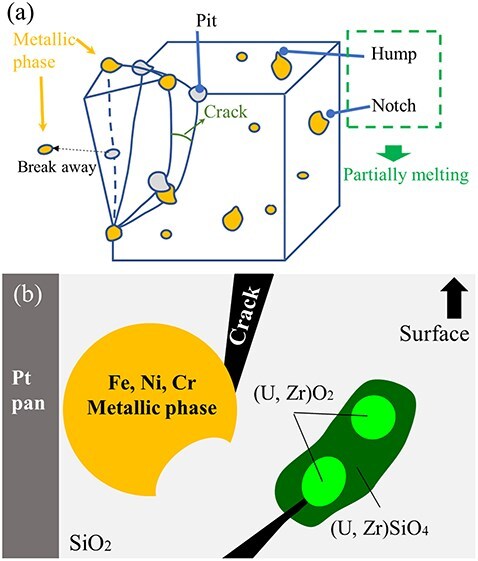
Schematic of the MCCI debris structures visualized by (a) 3D-FIB-SEM and (b) TEM. MCCI, molten corium–concrete interaction; 3D-FIB-SEM, 3D focused ion beam scanning electron microscopy; TEM, transmission electron microscopy.

The MPPM correction method is currently being improved for the analysis of fuel debris with more porous and complex structures, such as microvoids. Unfortunately, the latest 3D reconstruction showed two important features about the structures around the cracks in the fuel debris that should be considered. First, the crack tip reaches the (U, Zr)O_2_ core and stops there. Second, cracks propagate through the (Zr, U)SiO_4_/SiO_2_ interface and form many small voids. These open-tube structures can lead to environmental releases of Rn and ^220^Rn, which should be taken into account for the long-term stable storage and management of fuel debris. The morphological information, precipitate size and crystal system of the fuel debris crack tip revealed by the above 3D-FIB-SEM and TEM observations are schematically summarized in Fig. 11.

## Concluding remarks

In this study, we prepared Fe–ZrSiO_4_-based debris and (Zr, U)SiO_4_-fuel debris to simulate MCCI fuel debris in the Fukushima reactors and visualized the crack tips inside the MCCI debris at the nanoscale using 3D-FIB-SEM, WB-STEM and STEM–EDS measurements. The structural findings on the metallic phase and U and Zr oxide particles on the crack tips in the MCCI debris can be summarized as follows:

- A metallic phase with dimples on the surface of about 2–58 µm in diameter, mainly composed of polycrystalline body-centered cubic Fe, was formed at the crack tips.
- Inside the metallic phase, in addition to dislocations, microprecipitates of ZrO_2_ with a chemical behavior similar to that of UO_2_ were found to exist in Cr_2_O_3_.
- The dislocation density inside the metallic phase was 2.1 × 1014 m^−2^, which is three orders of magnitude higher than that of typical annealed Fe materials.
- The MPPM correction method allowed visualizing the (U, Zr)O_2_ core with a diameter of about 1–5 μm and the (Zr, U)SiO_4_ shell with a diameter of about 2–9 μm in the cross-sectional contrast, which were separated as cracks (brightness <35p) and charged-up regions.
- Numerous 500 nm cracks were observed at the boundary between the (Zr, U)SiO_4_ shell and the SiO_2_ region, and some of them propagated into the interior of the (Zr, U)SiO_4_ shell.
- Cracks did not penetrate into the (U, Zr)O_2_ core interior but propagated along the core–shell boundary. This could lead to the environmental release of Rn and ^220^Rn through the voids of the cracks.

The MPPM correction was developed in this study, improves the accuracy of drift corrections and reduces charge-up artifacts in tomograms obtained from nonconductive MCCI debris. Our in-house Python-based program for the MPPM correction is made publicly available in this paper. We hope that this study will pay the way for the future development of advanced imaging techniques for the microstructure elucidation of the Fukushima MCCI debris.

## Supplementary Material

dfaf005_Supp
